# Nutrition and Congruent Care Improve Wellbeing of Residents With Dementia in Slovenian Care Homes

**DOI:** 10.3389/fnut.2022.796031

**Published:** 2022-03-04

**Authors:** Anamarija KejŽar, Liljana Rihter, Jakob Sajovic, Gorazd Drevenšek

**Affiliations:** ^1^Faculty of Social Work, University of Ljubljana, Ljubljana, Slovenia; ^2^Department of Stomatology, University Medical Centre Ljubljana, Ljubljana, Slovenia; ^3^Laboratory for Cardiovascular Pharmacology, Institute for Pharmacology and Experimental Toxicology, Medical Faculty, University of Ljubljana, Ljubljana, Slovenia

**Keywords:** nutrition, wellbeing, dementia, elderly, person-centered approach, congruent care

## Abstract

**Introduction:**

Current nutritional strategies for people with dementia focus on nutritional diets and regimens, although in recent years congruent care for people with dementia has been increasingly recognized to improve their wellbeing. This includes consistency of care, respecting the variability of psycho-sociological factors, emphasizing the importance of participation in activities, and congruence with the individual's needs and capabilities. When applied to the nutritional aspects of care, it aims to empower people with dementia to have an active role in their care and during meals. Congruent care has previously shown promising results in improving the quality of life of residents, reducing the incidence of negative social interactions and daily intake of medicines.

**Methods:**

A mixed methods qualitative-quantitative study was carried out. Out of 102 residential care homes for the elderly in Slovenia, a non-random sample of homes was selected. Seven homes that have implemented congruent care and five who have not implemented it agreed to participate. Content analysis of the transcripts of focus group interviews was carried out, to establish how the congruent care model was included into their everyday practice of care for people with dementia. Qualitative comparative analysis was used to describe the differences in the practice of care between the two groups of homes, in the fields of nutritional and general care. Frequencies and assigned importance of statements relating to different aspects of nutritional care were statistically compared.

**Results:**

The introduction of congruent care improved the wellbeing of the people with dementia, as observed by caregivers. The homes that had implemented congruent care gave more attention to the food choice aspects of nutritional care (*p* = 0.0474, 95%CI_Congruent_ = 50.77–72.35%, 95%CI_Non−congruent_ = 27.65–49.23%), while the homes that had not were more attentive to the dietary intake aspects (*p* = 0.0067, 95%CI_Congruent_ = 22.79–44.74%, 95%CI_Non−congruent_ = 55.26–77.21%). In the homes for the elderly that had implemented congruent care, both caregivers and management reported that the frequency of use of *pro re nata* medication decreased, which is supported by the results of the linear regression (${\text{R}}^{2}{}_{\text{adjusted}}$ =78.4, *p* = 0.005), although the data available is limited.

**Conclusion:**

First, the people with dementia in the care homes that had implemented congruent care were observed to have improved in mood, attitudes toward eating and wellbeing, as reported by caregivers. Second, the implementation of congruent care was well received by the management and caregivers of the care homes. A model of implementation of congruent nutritional care for people with dementia is presented.

## Introduction

Due to increasing life expectancy, the number of people with dementia is rising sharply, making these disorders a global public health priority. The most common types of dementia are Alzheimer's disease, frontotemporal dementia, and dementia due to vascular disease. Worldwide, ~50 million people over 65 years are living with dementia, with 70% of them affected by Alzheimer's disease (1), a figure that is predicted to increase to 78 million in 2030 and 152 million by 2050 (2).

The common characteristics of dementia are impairment of memory and at least one domain of cognitive functioning, such as language, visuospatial skills, judgment or personality (3). Complex nutritional problems arise in dementia over the course of the disease, with the progressive decline in cognitive and behavioral functions ultimately leading to an inability to independently function in all aspects of life (4). During the onset of the disorder, impairments usually include difficulties in purchasing and preparing products, the preparation of simple dishes, forgetting to eat and drink, or eating multiple times a day. A decline in the sense of smell is also characteristic of the early stages of dementia (5). People with dementia may also experience increased appetite and rapid eating, and may repeatedly ask for food or exhibit compulsive eating (4).

As dementia progresses, so do the problems with eating—the inability to hold a spoon, to guide food to the mouth, impaired recognition of utensils or food, difficulty chewing or swallowing, loss of appetite or overeating, and the inability to drink from a glass. Especially in the later stages of the disease, malnutrition often occurs due to the refusal of food and fluids (6). Combined with the behavioral and psychological symptoms of dementia, such as depression, apathy and aggression, the capabilities of a person to continue eating autonomously are progressively impaired. It is therefore important to consider factors that positively affect the wellbeing of people with dementia at mealtimes and to consider their wishes and habits (7). One way to prolong the capabilities of people with dementia to eat on their own is by making meals as stress-free and enjoyable as possible. The general guidelines for the nutritional care of people with dementia are attractive and inviting meals, a daily routine and encouragement during mealtimes, variety in the menu, and respect for choice. The timing of meals and a calm and comfortable dining environment, as well as the food quality and suitability, are all important factors to consider (5, 8, 9).

In Slovenia, 59 public institutions and 43 concession providers offer long-term care (institutional care, day care and home care) with the capacity to care for 21,150 elderly people. The approaches with which care homes increase the wellbeing of their residents vary, but all adapt their processes of feeding to accommodate people with dementia. Some common adjustments that are made are in the consistency of the food, e.g. pureed and chopped food, and assistance with eating, such as spoon-feeding, in addition to the adherence to nutritional guidelines for the elderly. Aside from the nutritional aspect, many Slovenian care homes are implementing programmes of modern, integrated care for people with dementia to ensure the best possible quality of life. Since 2008, the household groups model has increasingly been adopted, featuring household units that provide individual care for 10–15 people with dementia (10).

Some public homes decided to take a further step, in addition to adopting the household groups model, and have introduced the congruent care programme, which includes a person-centered approach in all areas of life in a home for the elderly. Residents are treated as individuals and are equal partners in the process of care. It is personalized, coordinated and enabling, focusing on extending the autonomy of the residents and providing a supportive environment for when it becomes compromised (11). The implementation of congruent care, however, requires a large investment of both funds and time, as it is necessary to train all employees, from the management to the janitorial staff, in the methods of work according to the model. The process of implementation thus usually takes 5 years.

As one of the last areas of functioning that remain under the control of people with dementia, dedicating attention to the area of nutrition is of paramount importance in congruent care. In the spirit of congruent care, the individual's habits, desires, and possible dietary guidelines or restrictions need to be recognized and respected (12). Nutrition and the intake of food and drink can thus represent a key point of contact between the residents and caregivers, where the attunement of the caregiver to the person's wellbeing, wishes and habits places the resident at the center of care. To achieve this, caregivers strive to provide each individual with a meal in an environment where they feel safe and comfortable, and a meal they enjoy eating and can eat autonomously as long as they are able—with or without cutlery, mealtime extensions, snack corners and other adjustments (13, 14).

To more easily identify and evaluate the different areas of nutritional care that must be monitored and adjusted in everyday congruent care, Fostinelli et al. (5) suggest a taxonomy of three components: food choice (food preference and preparation); eating behavior (outcomes related to consumption, eating habits, eating disorders); and dietary intake (what is consumed, overall intake, specific nutrients). The authors further provide the theoretical framework that the three factors depend on (or are related to), i.e. the physiological (hunger, satiety, innate preference for sweet food) and psychological processes (learned food preferences, knowledge, motivation, attitudes, values, personality traits, cognitive processes, self-regulation). In addition to these, social factors also influence food consumption and must be considered, for example, a person eats more when eating in the company of others (15).

In addition to improving the quality of life of the residents, other health benefits were observed after the implementation of congruent care. Of specific interest is the observation by Galiana and Haseltine (16), who report a significant reduction in the daily intake of medicines and a significantly reduced weight loss in residents. Moreover, the authors also reported a significant reduction in adverse resident-to-resident interactions, less agitation of the residents at sundown, and a deeper connectedness of the caregivers to the residents. The less frequent agitation in particular was tied to the freedom of choice in eating habits, food items and other nutrition-related aspects of congruent care.

These results inspired us to design the present qualitative-quantitative study, where we aimed to examine how nutritional guidelines are included in the everyday care of the care homes that have adopted the congruent care model and those who have not. We also hypothesized that the use of PRN (lat. *pro re nata*, “as needed”) medication could be reduced in the care homes after the implementation of the congruent care model.

During our research, however, we found that there was little emphasis on dietary guidelines for people with dementia in either group of homes. Instead, the care homes organize their nutritional care not around guidelines, but according to their mode of operation, experience and capabilities. We therefore focused on the comparison and insight into how the two groups of homes approach nutritional care for people with dementia in general and in what ways they differ. The data on PRN medication use was very sparse, as we have discovered that these data are not routinely recorded and kept by the homes for the elderly. Thus only the data for PRN medication use in one home for the elderly was available, providing limited insight into the frequency of PRN medication use after the implementation of congruent care. Furthermore, we examined how care homes that adopted the congruent care model adapted their nutritional programmes for people with dementia. Based on these findings, we present a model for implementing congruent care in the field of nutrition of people with dementia.

## Methods

The study was a combined qualitative and quantitative study. The units of analysis were care homes, sampled from the population of 102 homes in Slovenia. The sampling in our study was targeted, meaning we chose 16 care homes, of which 8 had implemented the congruent care model for supporting people with dementia. We contacted the management of the homes and inquired whether they would be interested in participating in the current study, which would include focus group interviews being carried out with the management and the caregivers. From here on, the care homes are treated as statistical units, except for the analysis of medication consumption data, which was available only for one home, where the residents were treated as statistical units. From the group of care homes that implemented the congruent care model 7 responded, and from the second group 5 homes participated in our research. The frequencies of the response did not differ between the two groups, as tested by the Chi-square test of independence (${\text{χ}}^{2}{}_{\text{Pearson}}$ = 1.333, df = 1, *p* = 0.248). The two groups equally did not statistically differ in the number of participating homes (χ^2^ = 0.333, *df* = 1, *p* = 0.564). We can thus conclude that the sampling is most likely unbiased by group characteristics. Additionally, all of the involved homes follow the same procedures for the procurement of food and are subject to the same cultural norms and values.

Qualitative data was obtained with the use of four focus groups based on semi-structured interviews with management and caregivers in the 12 care homes in May and June 2021. All the focus groups were recorded with the agreement of the participants. They lasted between an hour and a half to two hours. The focus groups were transcribed and analyzed by qualitative analysis in a process where units (parts of sentences, sentences or whole paragraphs) of analysis were identified first and then open codes were defined for each part. Next, axial coding was performed (codes with similar meanings were grouped into categories and themes). Finally, relations were found between categories and/or themes that were relevant for our research questions (17).

We adopted the three-component taxonomy of dietary behavior of Fostinelli et al. (5), as it is designed to form a consensus in the levels of nutritional care needed for people with dementia. The three components (food choice, eating behavior and dietary intake) were thus used as the basis for our results, according to which the statements of the interviewees were grouped, and statistical analyses were performed.

Qualitative comparative analysis (18) was used to assess the attitudes of the caregivers and management of care homes toward the three main aspects of nutritional care; food choice, eating behavior and dietary intake. The analysis was carried out by first identifying all the statements made by the caregivers and management that at least somewhat related to any of the three categories (N_statements_ = 336). This pool of statements was then further narrowed down by the consensus of three researchers on whether the statement did in fact relate to any one of the three aspects (N_statements_ = 242). The statements were then divided into three groups, according to the aspect of nutritional care they pertained to, with some statements being included in multiple groups, as their contents related to two or all of the aspects (N_foodchoice_ = 74, N_eatingbehavior_ = 109, N_dietaryintake_= 67, N_statmentsinmultiplegroups_ = 6). Next, three researchers independently evaluated the statements with regard to the importance of the aspect of nutritional care to the home they conveyed, in a way blind to whether the statement originated from a care home with congruent care or without. The scores attributed were:

- 0 (We do not concern ourselves with this)
- 1 (We recognize this as a factor in the care for people with dementia)
- 2 (We are actively striving to pay attention to this factor in our everyday care and plan accordingly)
- 3 (This factor is exceptionally important to us, so we have gone to great lengths to ensure that needs associated with it are fulfilled)

Scoring was then averaged across evaluators, with the statements that had a minimum to maximum score difference of more than one (e.g. lowest score 1 and highest 3) removed from further evaluation. This was done in order to ensure that only statements where independent evaluators sufficiently agreed were further analyzed. For the food choice group of statements 11 statements were thus removed (14.9%), for the eating behavior group 17 (15.6%), and 7 statements (10.5%) for the dietary intake group.

Statistical testing was performed on the final scores, where the score of a statement was the mean of the three evaluators' scores. Testing of group differences was carried out with three separate Mann-Whitney *U* tests, with an alpha of 0.05 considered to be statistically significant. All tests were two-tailed. Mann-Whitney tests were used due to the non-normal distributions of all data, as assessed by one-sample Kolmogorov-Smirnov tests of normality (all *p* < 0.05), except for the congruent care homes dietary intake data, which also approached significance in the one-sample Kolmogorov-Smirnov test (*p* = 0.066). Two-sample Kolmogorov-Smirnov tests were used to corroborate the results of the Mann-Whitney U tests and to verify that the data came from the same distributions (p_foodchoice_ = 0.180, p_eatingbehavior_ = 0.972, p_dietaryintake_ = 0.99). The testing of the final scores was performed in order to determine if the two groups of homes differed in the expressed importance of the three aspects of nutritional care, where the null hypothesis was that they did not differ.

The group frequencies of statements, meaning whether a statement was given by the caregivers of a care home with congruent or non-congruent care, were tested by three separate binomial tests. These were performed in order to determine whether the two groups of homes differed in the number of statements given, with the null hypothesis being that they did not differ.

The amount of sedative or anxiolytic medication used as required (PRN) in people with dementia was available for one care home for 6 years after the implementation of congruent care. A linear regression model was used to assess the relationship between time (by year) and the number of events requiring this medication, in the years before and after the implementation of congruent care. The data fulfilled the criteria for linear regression (i.e. linearity).

SPSS v.25 (IBM, Armonk, New York, USA) and PRISM v.9 (GraphPad, San Diego, California, USA) were used to analyse and visualize the statistically tested data.

## Results

The response rates were 87.5% (seven homes) for the congruent care group and 62.5% (five homes) for the non-congruent group. Our sample encompassed 11.76% (12 out of 102) of all the care homes in Slovenia, meaning our sample is representative of the state of care for people with dementia in Slovenia at the institutional level. The care homes are located in most of Slovenia's major regions, giving a geographically sufficiently representative sample.

During the course of carrying out the research and after the analysis of the focus group results, it was discovered that there is not much focus on dietary guidelines for the care of people with dementia; instead, the caregivers and management of care homes attend more to the concept of nutritional care, regardless of whether the homes implemented congruent care or not. While implementation of specific nutritional guidelines for people with dementia, which would include nutrient ratios, amounts, etc., are lacking, the homes approach the problem of nutrition from a more comprehensive perspective, incorporating psychosocial aspects of human functioning into their care. This does not mean however, that no nutritional guidelines are adhered to, as care homes employ dieticians who construct the menus and ensure the nutritional quality of the food. The results merely point to the fact that no specific dementia-adjusted guidelines are observed.

We divided the results into two main sections: the first aims to describe how congruent care is implemented in practice, as well as the possible positive effects of such an approach to care. The second presents the differences between the homes that have implemented congruent care and those that have not. Both sections of the results first present representative statements obtained from the focus group interviews which illuminate the results of the qualitative analysis, which are then followed by limited quantitative analysis which helps to further the depth of analysis.

### The Results of Implementing Congruent Care

**Figure 1:**
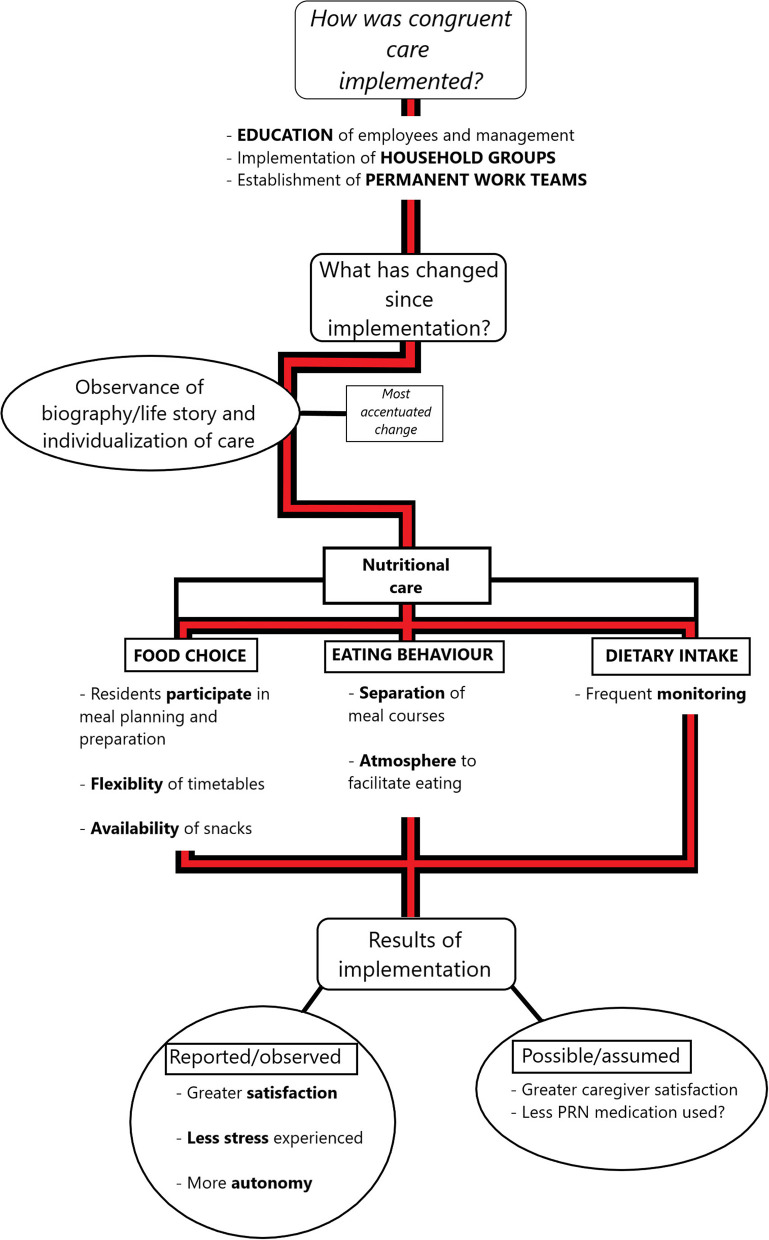
A summary of the results of implementing congruent care in caretaking of people with dementia. The red-and-black line shows the course of themes covered by the questions asked. The square boxes with the rounded edges show the three main areas of response, the ovals show important, highlighted results, and the emboldened text highlights keywords pertinent to answers that relate to specific topics.

The results of the focus group interview data pertaining to implementation of congruent care, the specific areas of dietary behavior that are focused upon, and the perceived effects of the implementation are presented in Figure 1.

#### How Was Congruent Care Implemented?

The results^1^ showed that the implementation of congruent care was based on three main factors. The first was the education of both caregivers and management in the model of congruent care, and their acquiring the communication and cooperation skills needed.

Some statements outlining this are quoted below:

- “… [We] *first enrolled management in these courses, and finally included autonomous work teams in them*.”
- “*The growth in the process of education, it is really important to ensure that what was learned becomes the new normal*.”

These statements show the trend observed, where the education of both management and caregivers is seen as crucial. Next, we see a desire to improve, and to base the improvements on expert opinion and established models. Finally, we see that the self-improvement of all employees is seen as important in ensuring that the changes implemented are successful.

The second factor was the establishment of household groups, an organizational approach, where the residents live in groups of up to 20 people, with each group having their own assigned permanent support by caregivers (such as housekeepers, nurses etc.). This was seen as another crucial step in implementing congruent care. The representative statements which show this include:

- “*Our 200 residents live in household groups, divided into 10 groups of 20 residents*.”
- “*The dynamics of life, of residence, are different in household groups*.”
- “*You just have to think of this as living with family. And we were able to implement this. Not only the household group, the whole unit can be as a family. We began this way of working some time before the implementation of congruent care in our residential units, where the residents and caregivers are permanent*.”

As these statements show, the household groups enable a more individualized approach to care, with deepening relationships between caregiver and resident.

The third factor was the establishment of permanent work teams, the importance of which is evident from the following statements:

- “*It's really important to have permanent teams of caregivers in household units*.”
- “ …*the key to effective care is really having staff permanently assigned to the resident's household groups.”*
- “...*the employees know each other, their own life stories, the life stories and residents themselves, as the residents know them in return*.”

The knowing of people and their personal characteristics, and the mutual familiarity of caregivers is important for creating a stable working and living environment that enables a focus on individual aspects of care. It also means that deeper relationships can be established. Having permanent staff in the household units is additionally viewed as a key component in effective care.

#### What Changes Have Been Made Since the Implementation of Congruent Care?

##### Life Stories and Individualization of Care

The most common change observed was the focus on life stories or biographies of people, where nutritional history and preference is recognized as one of the key aspects of care. This means that the caregivers in the care homes collected information on the preferred dishes and food of people before their admission into the home. These were further tied to life events (such as strong memories in childhood, or other important events, such as weddings) and used to construct individual plans of care for the residents. The importance of these life stories for the working process after the implementation of congruent care is evident from the following statements:

- “*The focus is very much on the individual, on their life story, what they like and what makes them feel good.”*
- “*We have many residents that walk around at night. For example, a female resident who used to be a nurse often comes to us at night when we are on call in the section of the care home for people with dementia and says: “Well, what now, who is going to sleep tonight? Me or you?,” to which I reply: “You go ahead and lie down, I'll be on call today. If the doctor calls, I'll call you, don't worry.” “Can I really go to sleep?” “Yes, go, sleep, I'll take care of it today.” Truly, this shows how important it is to know what lives our residents have lived and to know their life story.”*

Particularly from the second quote, the importance of using information from a person's life story to calm them down and take care of their concerns is evident. Without knowing that the person was previously a nurse, the caregiver might have been clueless as to what the female resident meant by who is going to sleep tonight and might have reacted differently, perhaps by attempting to return her to her own room or administering a sedative medicine.

The individual approach in the field of nutrition, specifically of food choice, is described in the following statement:

- “* …we make an effort to obtain information from the children of the residents about what their parents liked to eat before. Which dishes do they like, what don't they like? We then, of course, take this into account, enter it into their life story document and make that available to all the employees who work with that resident*.”

In turn, the knowledge of the persons' history and preferences allows for the formation of detailed, individualized nutritional and caretaking plans that provide the person with as much freedom, familiarity and support as they require. The importance of information transfer between different employees is also seen in the previous quote.

#### Nutritional Care

##### Food Choice

On the specific changes in the areas of food choice, eating behavior and dietary intake, changes to accommodate the specifics of people with dementia were made.

Pertaining to food choice, participating in meal preparation and menu planning was seen as very important. The way in which this is organized is seen in the following two statements:

- “*We organize a meeting with the residents once a week, so they can tell us what they want to eat*.”
- “*We include residents that are able enough in setting the tables before meals, placing the chairs around the tables, arranging the eating sets and giving out the food. After they have finished eating, they also help with putting the plates and cutlery into trolleys to be taken away*.”

Flexibility of timetables, meaning the residents are free to choose the time when they eat breakfast, for example, were identified as important, as well as the availability of snacks and finger foods. The snacks are used as an alternative method of food supplementation, ensuring the people eat enough calories and maintain their weight. The organization of this is illustrated by the following responses of caregivers:

- “*We leave them be; we do call out to them and invite them to join the meals, when the main mealtimes come round, but we see who comes and who doesn't. A resident might choose to first take a walk before a meal, as he prefers to eat alone, slowly, sometimes for more than an hour, while another might be punctual as a Swiss clock. We adapt to their wants and needs*.”
- “…*the introduction of this corner helped a lot, the residents liked it very much as well, the “Serve yourself” corner. There we always have fruit, in a form that is easy for them to consume, yogurts, warm drinks… For example, we have some whole and some sliced apples, as some residents are more likely to notice sliced apples and some more likely to see whole ones and help themselves. We also have grapes in bunches, cut into portions, so they are not too large. We also always have warm drinks, yogurt and cakes. The residents who normally eat less during meals often take advantage of this corner and eat something there*.”

We can see that in homes with congruent care, changes were made to enable people to make their own decisions with regard to food, the time of eating, and to provide them with alternative opportunities to take in enough calories and maintain weight. We can also see that the interventions are thought out with attention to the sensory and cognitive specifics of people with dementia (i.e. fruit that is in sliced and whole form). The maintenance of as much autonomy as possible is also seen as a priority, allowing extended independent functioning of people, where possible.

##### Eating Behavior

The two main changes that were made to accommodate eating behavior in people with dementia were made after adopting the congruent care model. The first was the separation of individual meal courses (e.g. soup, salad, main course, dessert), which allows people with dementia to focus on one dish at a time and prevents confusion due to too many different foods being available at once. It also prevents another issue with presenting all the meal courses at once, that is, people with dementia eating only the dessert and rejecting the other foods. The second change was the creation of an atmosphere appropriate for the consumption of food, removing distracting stimuli and encouraging the people to eat. A multisensory approach was taken in this case, including the use of smells (by cooking in a kitchen that is spatially connected by corridors to the rooms of the residents, allowing them to smell when meals are being cooked), verbal encouragement, help with cutting up or consuming the dishes, and controlling the noise levels (by turning down or turning off music). Sometimes preparation for the meal, such as assistance with dressing up and putting on makeup helps the people to partake in the meals with more motivation and encourages them to consume the food. The following statements outline the measures described above:

- [Separation of meal courses] “ …*we serve the food to every resident, while they are sitting at the table. This means we do not use trays for anyone, not that this is possible for people with dementia, but neither do we use trays with our other residents. The soup and main course, dessert or salad, whatever is on the menu that day, we place the next course in front of them after they have eaten the previous dish; we approach them so they can see us, then we give them some encouragement, so the eating is a bit easier. We want them to feel as much at home as possible. It's the same as at home, where we do not put everything we have for that meal on the table at once*.”
- [Creation of atmosphere] “*They come out of their rooms and start smelling what's cooking. It's a very positive feature. When food is prepared within the residential unit, it is very different to normal; everything smells of cooking in the unit.”*
- “ … *music has to be relaxing and quiet*.”
- “ … *it helps when they are all tidy and have their hair done in the morning*.”

##### Dietary Intake

Concerning dietary intake, more evaluation was implemented, while observing the individual life stories and data gathered by the staff. This allows for more rapid adjustments in the treatment of individuals. For example:

- “… *we make regular notes to use in the evaluation; that helps make sure nothing is missed when we evaluate our care*.”

#### Observed Effects of the Implementation of Congruent Care

Both caregivers and the management of care homes report that the people and their relatives are more satisfied with the food and treatment after implementation of congruent care. Caregivers also report higher levels of job satisfaction. Additionally, they report that they have had fewer negative interactions with the residents, in the sense that agitation of the residents is usually addressed in a different manner, with conversation, relaxing activities or gentle encouragement. This, coupled with reduced stress levels for both caregivers and the people with dementia, likely contributes to the observed reduction in the need for PRN medication, as reported by caregivers and management of the care homes. The greater autonomy of the people with dementia is achieved by the methods described in the results section pertaining to food choice. Some statements outlining these reported effects are presented below:

- [Satisfaction and stress reduction] “*We have seen a reduction in, I'll say, unwanted interactions with residents, stressful situations, and issues with residents, and this also benefits the caregivers.”*
- “…*Using congruent care shows in the levels of satisfaction that we survey every year, in residents, their relatives and our employees—all report over 90% of possible scores denoting satisfaction with care and work done*.”
- “… *not one of our caregivers would willingly revert to the old way of doing things*.”
- [Reduction of negative interactions and PRN medication use] “…*we have implemented the concept of personal monitoring, where we set out to teach our caregivers the techniques they need to calm the residents down, other than with medication. We have… the therapeutic discussion is at the forefront, touching them gently and sitting down with them, embracing them, calming them down with conversation. As was said, if they are hungry, we offer them something to eat, and they are immediately happier. We also use music therapy, adjusted for every individual. They often accept the headphones that we put over their ears, they listen to the music and calm down. The next measure is, of course, a safe space. They know this environment, they are free to come and lie down in the living room—if they feel safer with us, they don't have to stay in their rooms*.”
- “*PRN medication use has been reduced due to us being constantly in their company—we know them well-enough to adjust any medication needed as the situation changes, so we can reduce the medication when needed, not only increase it*.”

It is evident from these statements that the implementation of congruent care significantly improved the experience of caregivers, residents and their relatives.

##### Reduction in the Use of *Pro Re Nata* (PRN) Medication

**Figure 2:**
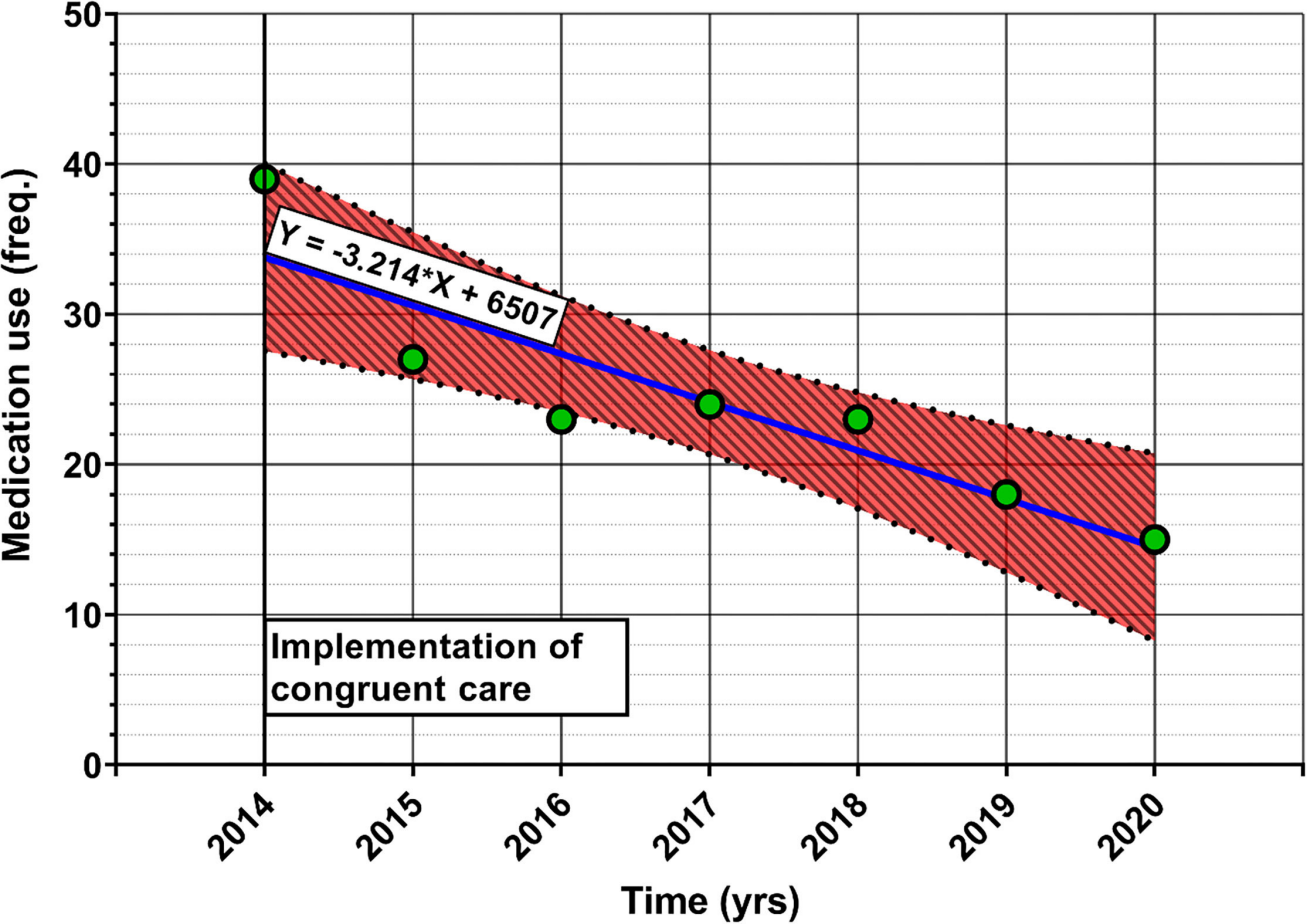
Linear regression of the frequency of PRN medication use (Y-axis) and time (in years, X-axis) since the implementation of congruent care in one home for the elderly. The red shaded area represents 95% CI for the regression line, with the blue line representing the regression line itself. The green dots represent individual data points. The equation on the regression line represents the equation of the line. The data is obtained from a congruent care home that decided to closely monitor the use of PRN medication after they began the process of implementation of congruent nutritional care.

We also hypothesized that a reduction in PRN medication use would be seen in the homes that implemented congruent care, but as the care homes mostly did not keep records of the PRN medication used, we were only able to obtain the data for one home that had implemented congruent care. We were unable to acquire any quantitative data from the homes that had not implemented congruent care on this topic, so between-group comparisons are impossible. The results of this analysis are a potential indicator of a relationship between the implementation of congruent care and PRN medication use, but are not grounds for any strong conclusions on the topic. Regardless, we present the results of a linear regression of PRN medication use since the implementation of congruent care, with Figure 2 presenting the results.

The linear regression model of time since the implementation of congruent care and PRN medication use proved to be significantly better than the null model (*F* = 22.75, *p* = 0.005). The time since implementation explained 78.4% of the variance (adjusted R^2^) in PRN medication use. These results show a significant association of the use of PRN medication and time since congruent care implementation, but warrants further research, with more variables and care homes included in the model to explain in more detail how the two variables are associated. From parameter B (or the slope parameter in the equation) of −3.214 [95% CI; −4.946–(−1.482)], however, we can assume, barring any confounding factors that are discovered in the future, that the implementation of congruent care could reduce the frequency of PRN medication administration by 3 (0.214) times per year, for at least 6 years after implementation.

#### Differences Between the Homes That Implemented Congruent Care and Those Which Did Not

**Figure 3:**
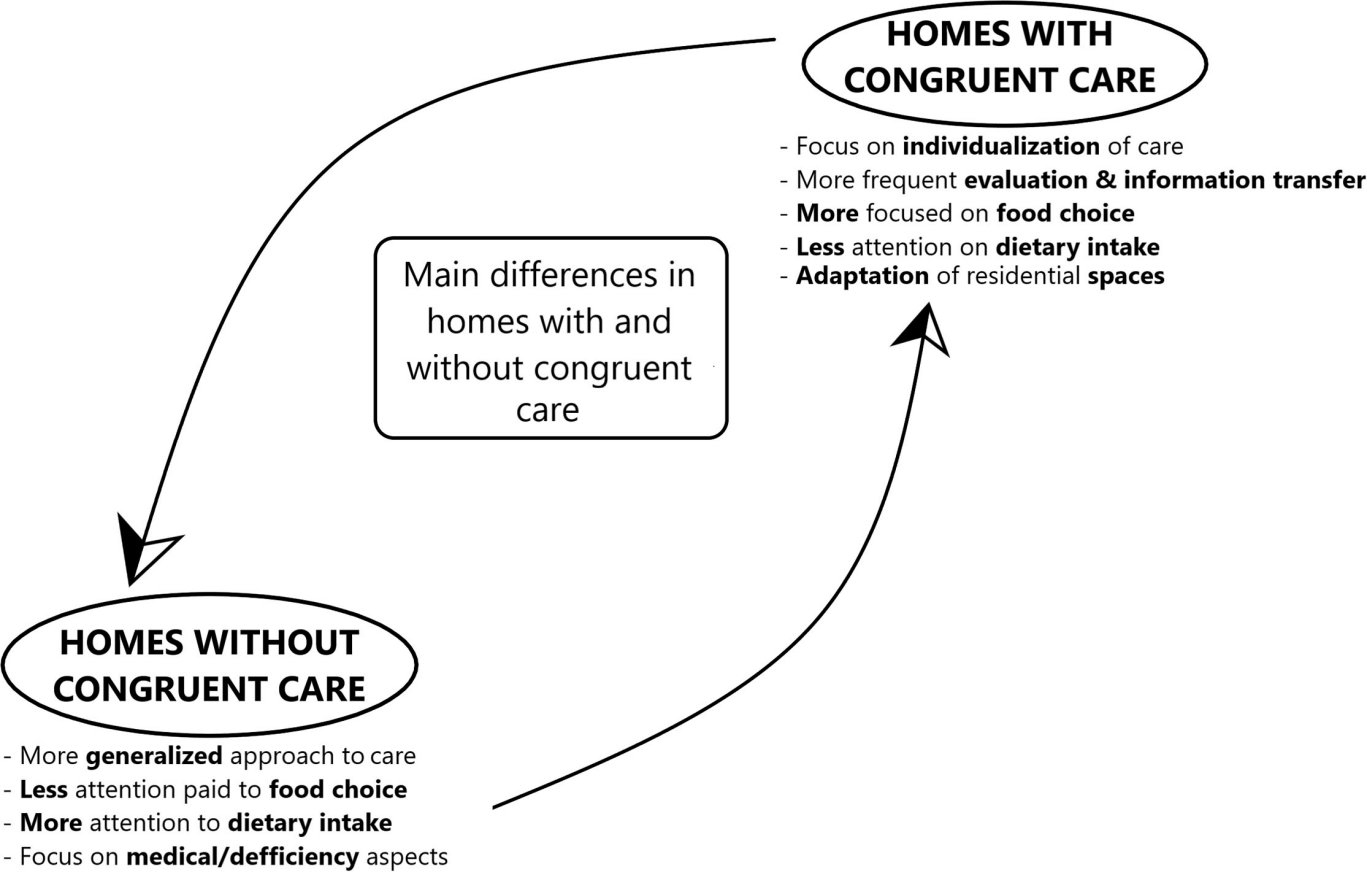
A summary of detected differences in the approach to care for people with dementia.

Figure 3 presents the results of a qualitative and quantitative analysis of data gathered from the focus groups that pertains to differences in care for people with dementia in Slovenian care homes.

During the analysis of the transcripts of the focus group recordings it became evident that the care homes that had not implemented congruent care took a more general approach to nutritional care than the homes with congruent care. In summary, both caregivers and management reported that less attention is paid to the individualization of meals and preferred foodstuffs, which can be seen in the following statements (Table 1).

**Table 1:** Selected statements on the approach to nutritional care.

	**Care homes with congruent care**	**Care homes without congruent care**	
Approach to nutritional care		“*My residents would like to eat beans, potatoes and cabbage all the time. That's the fact of our location. So we have a joint group of caregivers and residents, with the head nurse and cook, to make a menu for each month.”*	“*Once every three months all the cooks and dieticians meet and make the menu and so on.”*
		“*Absolutely, we take mealtimes into account. We also offer a choice, as my colleague said. Everyone can choose what they eat for breakfast—will they eat a spread of one sort or another, maybe that one, will they eat salami, an egg, maybe three eggs?”*	“*…we adjust, so the food that the people with dementia find difficult to eat, we offer something else, as with the other residents”*
		“*We honor their wishes, so they can sleep in and join the meal later.”*	“*During the day the activities are structured in the same way as for the other residents, but they have a timetable, because we find it very important to provide a routine to the residents with dementia. This means when there is breakfast, it is breakfast, and after that we have activities. When it's time for coffee it's time for coffee, maybe we offer some biscuits. Later we also have lunch, afternoon and morning snacks, as well as dinner.”*

On the other hand, more attention is given to the medical and deficiency aspects, even when individualization of nutritional care is present, meaning that in homes with congruent care there is a focus on the individual so they can quickly observe if a resident does not want to eat. Yet in non-congruent homes (more) focus of care is evident when people already face malnutrition or other medical complications. These differences are shown in Table 2.

**Table 2:** Selected statements on the focus on individualization in care homes, by group.

	**Care homes with congruent care**	**Care homes without congruent care**	
Focus on individualization	“*Absolutely, if a person doesn't want to eat anything that is available, we always find something they like or we make something especially for them, so they can eat it that day. If they want something else, we always try to fulfill that wish.”*	“*…in people where these difficulties occur, when they can no longer swallow normally, they don't know how to swallow the food. Then we consult with the dietician, or use our good practices from the past, which allow us to ensure they get the calorific intake they need.”*	
		“*We try to fulfill their wishes, so they can sleep in or come for a meal later, not at a pre-set time, as we used to do.”*	“*We do individualize care, for example, in people with dementia, they often start to choke on their food.”*
		“*We try to be really focused on the wishes of the residents in the area of nutrition. But, of course, we cannot ignore the doctors' prescriptions.”*	“*…individually, we then, one way or another, individualize care, if we find that someone does or doesn't want something, or when we find that a person is malnourished or in need.”*

The care homes with congruent care also gave more detailed descriptions of evaluation and information transfer between caregivers in different roles, with such descriptions being absent from the answers of homes without congruent care.

**Figure 4:**
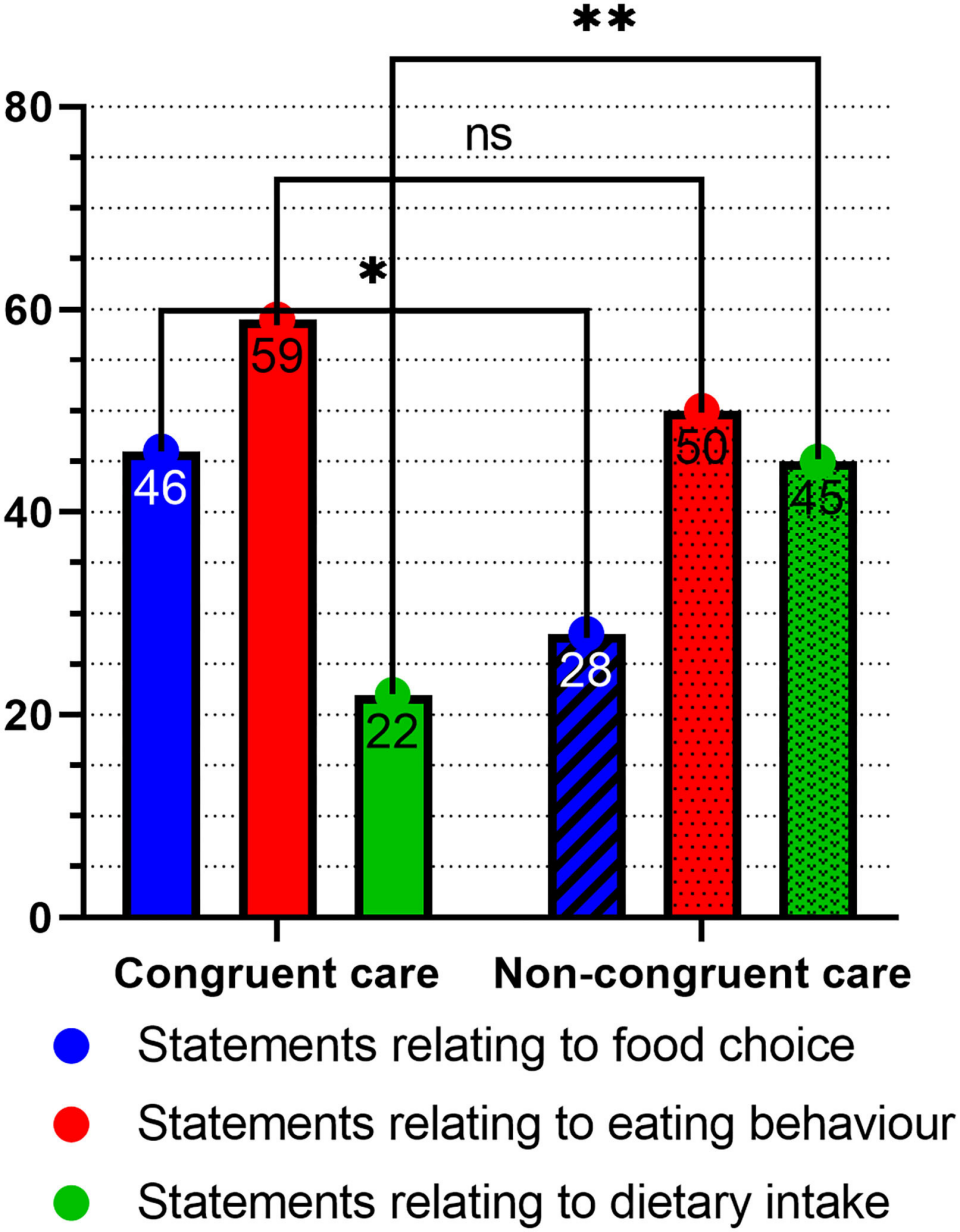
Differences in frequency of statements relating to a specific area of nutritional care for people with dementia. Three separate binomial tests were run to determine the between-group differences. The ^*^ symbol denotes a statistical significance of < 0.05, while the ^**^ symbol denotes a statistical significance of < 0.01.

These results are corroborated by the analysis of the number of statements given by each group of homes on the specific topic of nutritional care for people with dementia (Figure 4).

The results of the comparisons with binomial tests are: for food choice *p* = 0.0474, 95%CI_Congruent_ = 50.77–72.35%, 95%CI_Non−congruent_ = 27.65–49.23%; for eating behavior *p* = 0.4437, 95%CI_Congruent_ = 44.79–63.18%, 95% CI_Non−congruent_ = 36.82–55.21%; and for dietary intake *p* = 0.0067, 95%CI_Congruent_ = 22.79–44.74%, 95%CI_Non−congruent_ = 55.26–77.21%. All confidence intervals are reported as % of all statements.

As the frequency of mentioning a specific topic can be construed as that topic being more in the forefront of a person's mind, we can conclude that the caregivers and management of the care homes that have implemented congruent care pay more attention to the food choice aspects of care and less to dietary intake, while the reverse is true for the caregivers and management of homes with non-congruent care.

The results of the analysis of the statements, evaluated on the importance attributed to specific topics of nutritional care, were non-significant. From this, it follows that we cannot say that the two groups of homes differed in the assigned importance to each of the three aspects of congruent care: food choice, eating behavior and dietary intake.

## Discussion

The present study was designed and carried out to investigate how the implementation of congruent care affected adherence to dietary guidelines for people with dementia. In the process of the research, it was found that neither group of care homes adhered to any specific guidelines. Instead, a psychosocial approach to nutrition was favored, with both groups of homes assigning equal importance to the food choice, eating behavior and dietary intake aspects of nutritional care. The differences between the groups of homes that have implemented congruent care and those that have not are as follows (Figure 4; Tables 1, 2):

1. The care homes with congruent care are focused more on the food choice aspects of care, while more attention is given to dietary intake by the homes with non-congruent care (Figure 4).
2. The focus of the individualization of care, while present in both groups of homes, is different. The group without congruent care focuses more on the medical aspects, and attention to dietary intake and care is evident when people already face malnutrition or other medical complications, while homes with congruent care favor aspects which improve quality of life (Table 2).
3. The approach to nutritional care is more generalized in homes with non-congruent care, while more accommodation of individual preferences and needs is made in homes with congruent care (Table 1).

The finding from the data on PRN medication use of one care home with congruent care warrants further investigation by future studies. Although the management and caregivers of the homes with congruent care reported that a reduction in the use of PRN medication is present, which concurs with the results of the data analysis of the one home, the limited amount of available records bars us from making strong conclusions on the topic.

Example statements on the reduction in medication use:

- “*PRN medication use was reduced, because we, the caregivers, know the residents, which means we are able to recognize a deterioration in the mental state of a resident, which enables us to attempt to steer them away from a difficult situation by talking to them*.”
- “*Additionally, daily medication use is reduced because we are there, with them all the time, and we know them well-enough to be able to reduce, not only increase, the dosage of medication when any changes are detected*.”

The unfortunate fact that most homes do not keep detailed records of PRN medication use should be overcome in the future, as this is an important variable to follow. This is recognized by the caregivers and employees of care homes, as it was stated during the focus group interviews that:

- “…[This] *has to be worked out, how to follow this* [the PRN medication use] *effectively*.”
- “*As for PRN medication use in care homes, a resident pharmacist post should be created and filled in homes in Slovenia, …as someone who has knowledge of drug interactions and would closely follow medication use could surely reduce its use by 20–30%*.”

Following on from these results, we recommend that the effects of the implementation of congruent care on PRN medication use is confirmed by future studies, and that care homes keep detailed records of their PRN medication use, where the data is not only gathered but also collated into useful information that can be analyzed and interpreted. This will enable more transparency in PRN medication use and easier adjustment when the data of an individual person is considered.

Some benefits of the implementation of congruent (nutritional) care are obvious. For example, a satisfaction level of over 90% in residents, relatives and caregivers was seen in care homes with congruent care, while no such reports were given by non-congruent homes. This is in line with previous results on this topic, by Galiana and Haseltine (16), who reported a similar level of satisfaction with care in the care homes they analyzed. Moreover, the fact that several caregivers of homes with congruent care stated that they would not willingly revert to the previous mode of work is salient to this discussion and supports the effectiveness of congruent care.

The nutritional aspects are recognized as crucial in the care for people with dementia, both in the extant literature (4, 16) and in the opinion of the caregivers interviewed in our study;

- “*As we know, food is one of the primary human needs, which remains important until the day we die, and as such contributes to quality of life*.”

Regardless of the importance of food and nutrition in everyday care, the management and caregivers of the care homes that participated in our study expressed a concern that there is a lack of training courses or other options for education in the field of nutrition that are focused on these aspects of care for people with dementia.

**Figure 5:**
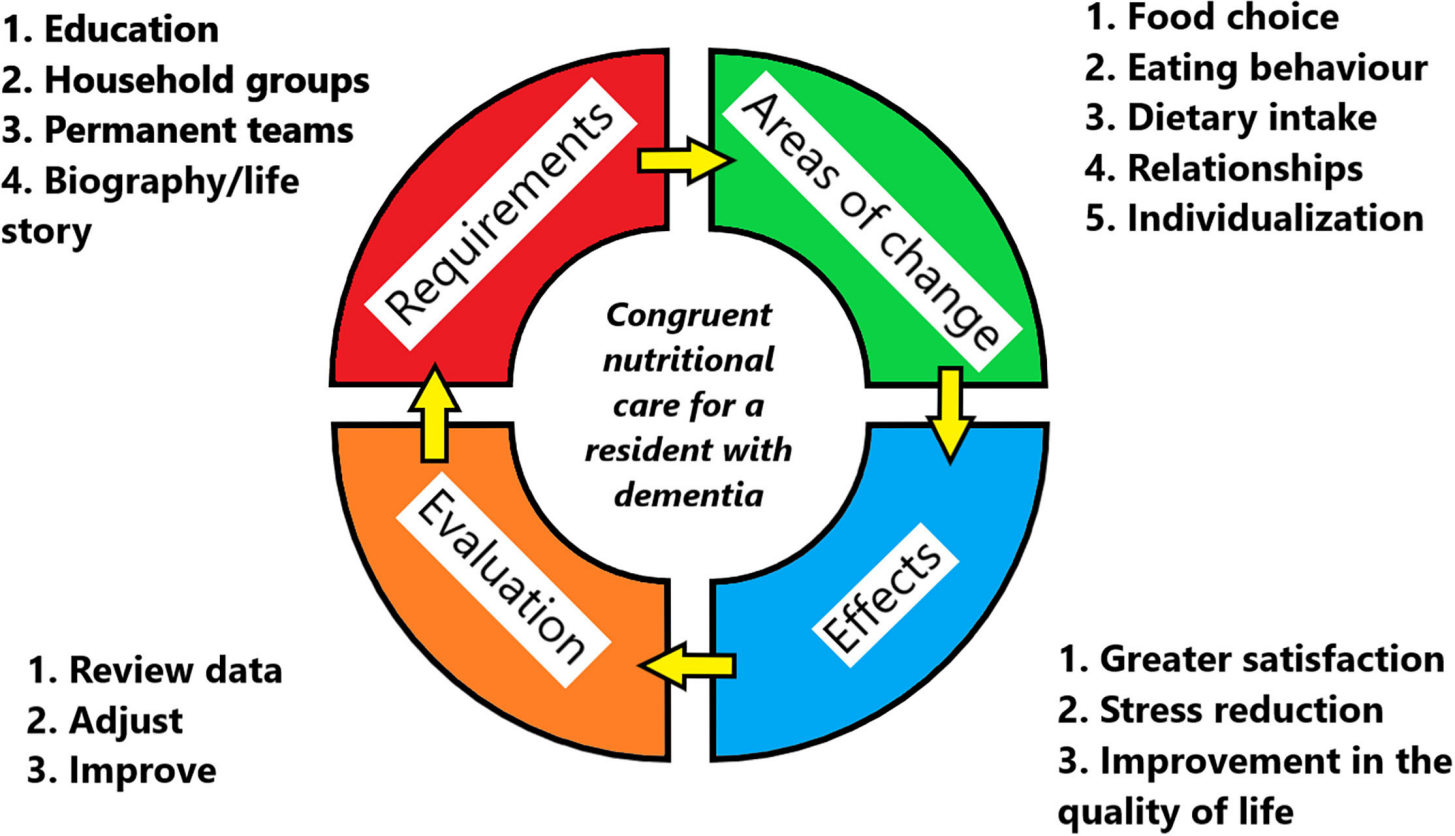
The proposed model of the implementation of congruent nutritional care for people with dementia. Each quarter represents a crucial field of implementation, beginning with the requirements.

To address this issue, we propose a four-part model for the implementation of congruent nutritional care for people with dementia, which synthesizes the experience of the staff and management of the care homes that adopted congruent care, and the current theoretical framework for nutritional care for people with dementia (4, 5, 16), presented in Figure 5.

Beginning with the requirements (Figure 5, red panel) for implementation, education for both caregivers and management is crucial when congruent nutritional care is to be implemented in care homes. Some care homes that have implemented congruent care have solved the issue of the shortage of available educational programmes by hiring professional chefs or by organizing internal education courses led by their resident dieticians in order to provide information for the caregivers on food preparation and dietary specifics.

Another important factor is the availability of space to reorganize the care and living arrangements into household groups. The residents ideally live in groups of 10 to 15 people in these household groups, with the care required being provided by the caregivers within the household. Each household has a permanent “housewife” assigned, who is in direct everyday interaction with the residents and acts as a “point of information” about the household group. Most of the daily life in these household groups revolves around a central kitchen and living room, where people can mingle and socialize. The food is also prepared there, which enables the residents to more easily help in its preparation, and allows for the smells of cooking to spread around the household, improving their interest in food. This way of organizing the care is important, as it allows the elderly people to retain the greatest possible resemblance of normal daily life, and builds the feeling of homeliness and familiarity, which has a calming effect on the residents (16). Moreover, it provides a constant point of contact between the residents and food preparation that fosters the retention of autonomy in the area of nutrition.

To support people with dementia in maintaining their autonomy as much as possible, permanent work teams are crucial (also see the Results section How Was Congruent Care Implemented?). Permanent work teams allow the formation of deeper personal connections, trust and mutual understanding between the residents and caregivers that enables easier, quicker and smoother adjustment of care to the needs of people with dementia.

A system of information gathering is the final requirement for the implementation of congruent nutritional care for people with dementia. To construct the life stories of their residents, caregivers should obtain information from multiple sources, ranging from the residents themselves and information from medical records to interviews with family members and their own observations. The caregivers and other staff of the care homes must have access to the life stories, which allows them to attune to the resident based on their past, and for a deeper understanding between the caregivers and the resident to develop. This is beautifully illustrated by the statement of a caregiver in a care home that had implemented congruent care, which describes how a female resident who used to be a nurse wandered the halls of the home at night and the way in which this issue was resolved (see Results section Life Stories and Individualization of Care). From the standpoint of nutrition, by knowing the preferences of people with dementia, their life stories and ancillary information, the caregivers are once more able to more efficiently adjust to the needs and wants of the residents (19, 20).

There are some areas where the methods of providing nutrition must change from the regular modus operandi (Figure 5, green panel). The three aspects of nutritional care for people with dementia (5) should be considered when providing care, i.e. food choice, eating behavior and dietary intake. The purpose of congruent care, in nutrition or otherwise, to maintain autonomy, dignity and to provide compassionate treatment to the residents (11) should always be in the forefront of the mind when making a plan of care for people with dementia.

Thus the food choice aspect should be focused on, but not to the extent that the other two aspects are neglected. This is also seen in the results of the analysis of assigned importance (Results section Differences Between the Homes That Implemented Congruent Care and Those Which Did Not) and the frequency of statements (Figure 4), where no differences were found in the importance that the two groups of homes expressed with regard to the three aspects, but the homes with congruent care exhibited a greater amount of attention to the food choice aspect (Figures 3, 4). With regard to the results of the differences in the number of statements made on the topic of dietary care, this is most likely due to the caregivers and management of the homes without congruent care operating differently, focusing more on the traditional medical modes of nutritional care and individualization (Table 2). The focus on food choice enables the residents to maintain their autonomy and is a key part of the individualization of care (8), and so should be given attention, allowing the person to participate in meal preparation and menu planning. Mealtimes should be flexible, to allow people with dementia to maintain their own rhythm of daily activities. To provide multiple opportunities to take in calories during the day, snacks should be made available, with a “serve yourself” corner being effective (Figure 1, Results section Food Choice).

With regard to eating behavior, it is important to recognize that meals are more than just an intake of food. While their primary function is to provide the body with the required nutrients, meals are important social events that are inextricably linked to the local culture (21, 22). To maintain the social function of meals, which can benefit the residents by reminding them of some of their core memories when congruent with their cultural background, the food-associated traditions of individuals should be understood. These are most often shaped by the individuals' upbringing and the social environment in which the food is served and eaten (23–26). The knowledge of this, linked to the initial data gathering, enables the creation of an appropriate atmosphere for the ingestion of food. Further important factors that influence the wellbeing of people with dementia at mealtimes are sensory: smell—preparing food together with a person with dementia or in front of them; sight—arrangement, food presentation, use of color contrasts, lighting; taste—preparing food that a person with dementia has known from an early age; and hearing—providing a calm environment during mealtimes. As no two people are alike in their preferences for food and its aesthetics, it is of great importance that the subject of nutrition is approached with individualized care and that appropriate strategies for preserving quality of life are adopted in the care of people with dementia (27–29). Considering the specifics of dementia, meal course separation, i.e. serving the soup, main course and dessert consecutively, should always be applied (9) (Figure 1, Results section Food Choice). The dietary intake of residents should be regularly monitored and included in their biographical data, to enable easy overview and adjustment of nutrition, as required. The recognition of the importance of dietary intake in congruent care in the statements given by the caregivers and management of homes with congruent care can be found in the Results section What Changes Have Been Made Since the Implementation of Congruent Care?.

The combination of permanent work teams, knowledge of the people and a compassionate approach thus give rise to a person-centered approach to care, but additionally, one that is based on a genuine, affectionate and deep relationship between the caregivers and the residents, which enables the best quality of life and care for the elderly with dementia (12, 16).

The effects that can be expected (Figure 5, blue panel) after the implementation of congruent care and its application to nutrition are a reduction in stress and adverse social interactions, an increase in satisfaction with care of residents and their relatives, an increase in the job satisfaction of the caregivers (11, 16) (Results section What Changes Have Been Made Since the Implementation of Congruent Care?, Figure 2). A reduction in the need for PRN medication was observed on our data, as well as reported by the management and caregivers of the homes for the elderly. This association should be verified to clearly establish the relationship between the implementation of congruent care and the need for PRN medication. To ensure that the implementation is successful and has the desired effect, the satisfaction of the residents, their relatives and the caregivers should be monitored (Figure 5, orange panel). Additionally, detailed records of medication use should be kept, alongside dietary intake data and any events of note, such as data on the occurrences of adverse social interactions. The gathered data should be regularly evaluated and the care adjusted if the desired effects are not seen, in order to continuously improve and evolve the services provided.

To conclude, the implementation of congruent care has the potential to reduce stress and increase wellbeing, while improving both the living conditions of the residents and the working conditions for the caregivers and other employees of the care homes. A congruent approach to nutritional care can thus assist the caregivers in helping our elderly people with dementia retain the dignity and autonomy they deserve.

## Data Availability Statement

The datasets presented in this study can be found in online repositories. The names of the repository/repositories and accession number(s) can be found below: Figshare repository; https://doi.org/10.6084/m9.figshare.16817476.

## Author Contributions

GD, LR, and AK conceptualized and designed the study. LR and AK gathered the data. JS, LR, and AK analyzed the data. All authors participated in the writing of the manuscript. All authors have read and approved the final version.

## Funding

JS acknowledges the funding by the Slovenian Research Agency programme P3-0293(B). LR acknowledges the funding by the Slovenian Research Agency project J5-2567. GD and AK acknowledge funding by the Erasmus + Capacity Building in Higher Education 2020 nEUROcare project, ref. 618596-EPP-1-2020-1-SE-EPPKA2-CBHE-JP.

## Conflict of Interest

The authors declare that the research was conducted in the absence of any commercial or financial relationships that could be construed as a potential conflict of interest.

## Publisher's Note

All claims expressed in this article are solely those of the authors and do not necessarily represent those of their affiliated organizations, or those of the publisher, the editors and the reviewers. Any product that may be evaluated in this article, or claim that may be made by its manufacturer, is not guaranteed or endorsed by the publisher.

## Footnotes

^1^As the interviews were carried out in Slovene, only representative statements were translated into English and were kept as literal as possible to maintain the meaning and provide the closest approximation of the originals. For a full translation, please contact the corresponding author.
